# Single stage: dorsolateral onlay buccal mucosal urethroplasty for long anterior urethral strictures using perineal route

**DOI:** 10.1590/S1677-5538.IBJU.2015.0184

**Published:** 2016

**Authors:** Vikram Prabha, Shishir Devaraju, Ritesh Vernekar, Murigendra Hiremath

**Affiliations:** 1 Department of Urology; KLE University’s JN Medical College, Belgaum, India; 2 Department of Nephrology KLE University’s JN Medical College, Belgaum, India;; 3 Department of Studies in Microbiology & Biotechnology, Karnatak University, Dharwad, India

**Keywords:** Urethral Stricture, Mouth Mucosa, Reconstructive Surgical Procedures

## Abstract

**Objective:**

To assess the outcome of single stage dorsolateral onlay buccal mucosal urethroplasty for long anterior urethral strictures (>4cm long) using a perineal incision.

**Materials and Methods:**

From August 2010 to August 2013, 20 patients underwent BMG urethroplasty. The cause of stricture was Lichen sclerosis in 12 cases (60%), Instrumentation in 5 cases (25%), and unknown in 3 cases (15%). Strictures were approached through a perineal skin incision and penis was invaginated into it to access the entire urethra. All the grafts were placed dorsolaterally, preserving the bulbospongiosus muscle, central tendon of perineum and one-sided attachement of corpus spongiosum. Procedure was considered to be failure if the patient required instrumentation postoperatively.

**Results:**

Mean stricture length was 8.5cm (range 4 to 12cm). Mean follow-up was 22.7 months (range 12 to 36 months). Overall success rate was 85%. There were 3 failures (meatal stenosis in 1, proximal stricture in 1 and whole length recurrent stricture in 1). Other complications included wound infection, urethrocutaneous fistula, brownish discharge per urethra and scrotal oedema.

**Conclusion:**

Dorsolateral buccal mucosal urethroplasty for long anterior urethral strictures using a single perineal incision is simple, safe and easily reproducible by urologists with a good outcome.

## INTRODUCTION

Urethral stricture is a common disease encountered by urologist. Exact incidence in Indian population has not been reported. Reconstruction of long and complex anterior urethral strictures is technically demanding. Long anterior strictures with dense focal narrowing and scarred, extremely narrow urethral plates, fistula or infection are best managed with staged procedures (1, 2). Those with a salvageable urethral plate are being increasingly managed with a single stage repair using genital or non-genital tissues grafts/flaps (3, 4). Since Suprechko’s first description of buccal mucosa used as a graft in 1886, it has become the tissue of choice for urethral reconstruction (5). Its popularity can be credited to extensive work by Braca and Barbagli. It is readily available and easily harvested with minimal donor site morbidity. Buccal mucosa is hairless, has a thin, elastin rich epithelium giving it excellent handling characteristics and a highly vascular lamina propria, which facilitates harvesting and imbibition. The ideal location for BMG onlay has been debated for quite some time. There is now adequate evidence that dorsal onlay has an edge over the ventral onlay technique, especially in the penile urethra (5-7). Recently Barbagli and Kulkarni have proposed one-sided mobilization of the urethra with sparing of central tendon of perineum and dorsal anterior/lateral placement of the BMG in order to preserve the blood supply to the urethra and neuro-vascular integrity of the bulbospongiosus muscle respectively (8, 9).

We present our experience single stage urethroplasty with dorso-lateral onlay of BMG for long strictures of anterior urethra approached through a perineal incision.

## MATERIALS AND METHODS

The study was conducted between August 2010 and August 2013. Approval was taken from the hospital ethical committee. Patients who presented to us with anterior urethral strictures (>4cm measured on RGU) were included in the study. Each patient was evaluated by detailed history, physical examination, uroflow with post void residual urine, RGU and VCUG, and other routine investigations necessary for surgery. A suprapubic catheter was placed pre-operatively in those presenting with acute retention of urine and/or with altered renal parameters. The cause of stricture was Lichen sclerosis in 12 cases (60%), instrumentation in 5 cases (25%), and unknown in 3 cases (15%).

Exclusion criteria were previous failed urethroplasty, urethral abscess, urethral fistulas and a scarred and unsalvageable urethral plate.

Uroflowmetry and measurement of post-void residue was done at 1 month, 3 months and 6 months after surgery and every 6 months for the first 3 years thereafter. Those who had a recurrence of voiding symptoms with an objective evidence on uroflow study underwent imaging and/or cystoscopy to identify the site of re-stricture. These cases were considered as treatment failures.

**Figure 1:**
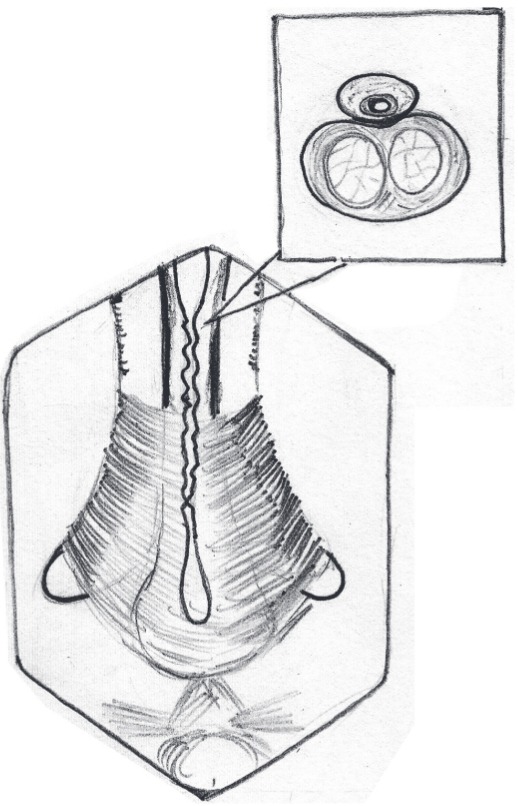
Dissection up to level of bulbo spongeosis with long stricure in penile and bulbar urethra.

Operation was performed under general anesthesia with nasal intubation. Two teams worked simultaneously, one at the donor site and other at the recipient site. Urethroscopy was performed using a 6-7.5Fr semi rigid (Karl Storz) ureteroscope and a hydrophilic (Terumo) guide wire was passed into the bladder. A 5fr ureteric catheter was guided over it and the ureteric catheter was secured with a stich on the glans. A midline perineal skin incision is made; the bulbar urethra is exposed, preserving the midline tendon of the perineum and bulbospongiosus muscle (Figure-1). The involved bulbar urethra is dissected off the corpora cavernosa on the left side, so as to leave the right half attached and preservation of its lateral blood supply.

**Figure 2:**
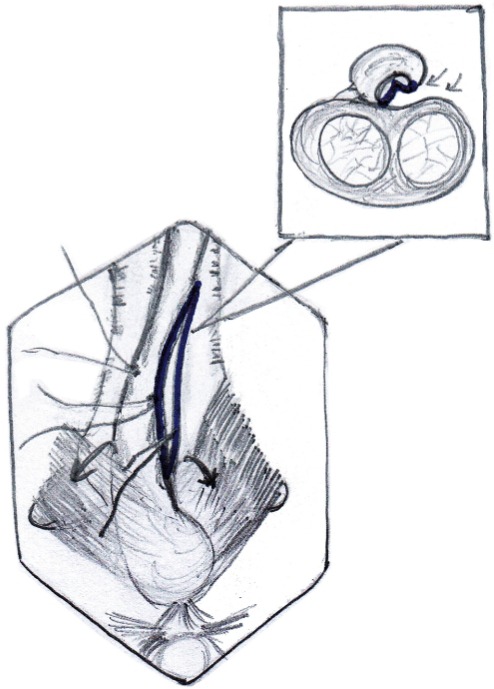
Dissection of right half of copus spongiosum off the copora cavernosa dorsally and opening of the stricture segment Note: Bulbo spongiosus muscle being retracted down to facilitate exposure.

The penis is invaginated into the perineal incision and the involved segment of penile urethra is similarly dissected of corpora cavernosa along the left side. On the left side urethra is partially rotated and the dorso-lateral surface is incised exposing the lumen (Figure-2). The incision is extended for about 1cm beyond the stricture segment at both ends. The proximal and distal lumen is calibrated to ensure adequate patency. In case of strictures extending up to the external urethral meatus, a dorsal meatotomy is performed from the meatus, through the urethra inside the glans, connecting it to the dorso-lateral incision in the distal penile urethra.

The buccal mucosa is harvested from the inner cheek (one or both sides, depending on the length required). The inner cheek from just inside the labial angle up to the retromolar trigone is marked, keeping 0.5cm away from the opening of the Stensen duct, to obtain a buccal graft of 2.5-3cm width and 6-7cm length. We use a 26 gauge needle to infiltrate dilute (1:200000) adrenaline under the marked portion of the mucosa. The edges are incised, 2 stay sutures are placed at the distal corners of the graft using 3-0 chromic catgut, for traction. Once the graft is harvested, the raw area is allowed to epithelize secondarily. The graft is defatted, trimmed to an appropriate shape and used as an onlay. We do not perform a primary closure of the mucosal defect.

The buccal mucosal graft is trimmed to an appropriate size and is spread and fixed (quilted) over the exposed half of the corpora. The edges of the graft are sutured to the corresponding edges of the opened urethral lumen using 4-0 polygalactin sutures (Figures 3a, 3b and 3c) over a 14Fr silicone Foley’s catheter. In those cases with external urethral involvement, the dorsal meatotomy incision allowed us to widen the narrow meatus/fossa navicularis region and draw the graft in through the glans from the distal urethrotomy and place it right up to the tip of the external meatus (Figures 4a and 4b). After completion of anastomosis, the wound is closed in layers (Figure-5). The periurethral catheter is left in-situ for 3-4 weeks.

**Figure 3a:**
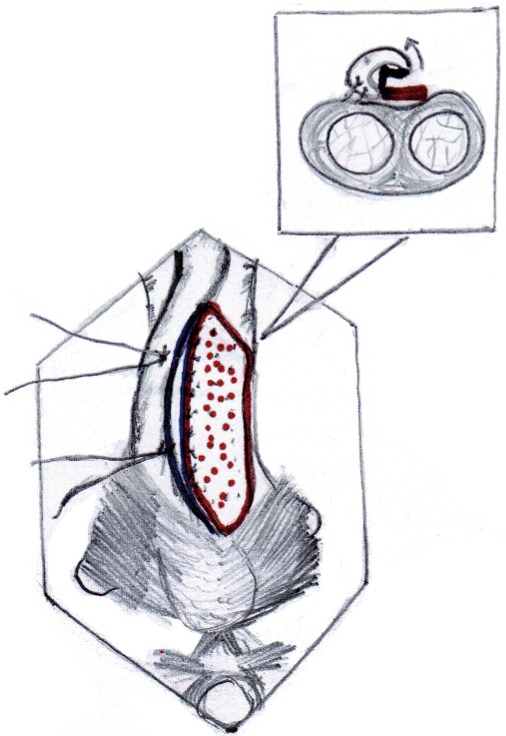
Buccal mucosa graft placed in position by quilting over the tunica of corpora cavernosa.

**Figure 3b:**
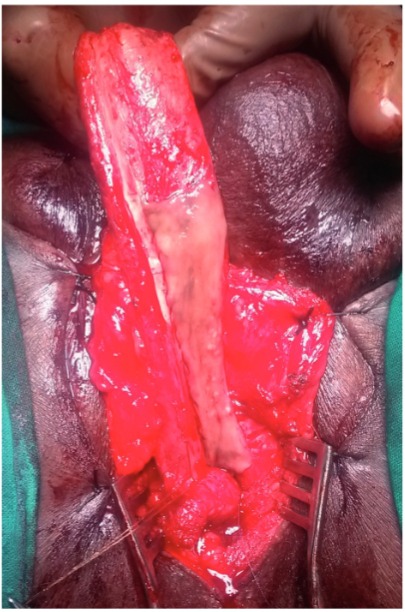
Intra operative image showing placement of buccal mucosal graft.

**Figure 3c:**
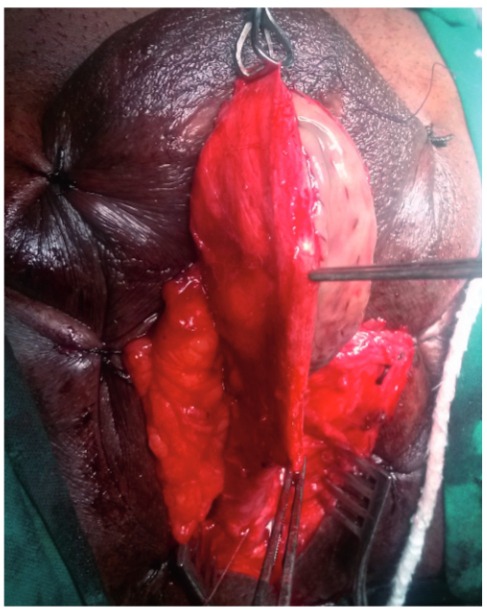
Intra operative image showing completed graft placement. Note: Partial mobilization of corpus spongiosum.

**Figure 4a:**
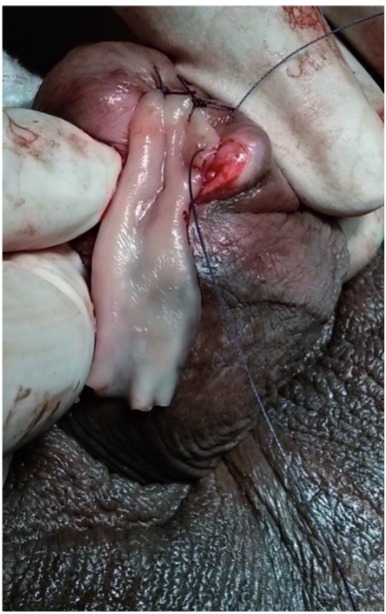
Dorsal meatotomy and graft placement at the external urethral meatus.

**Figure 4b:**
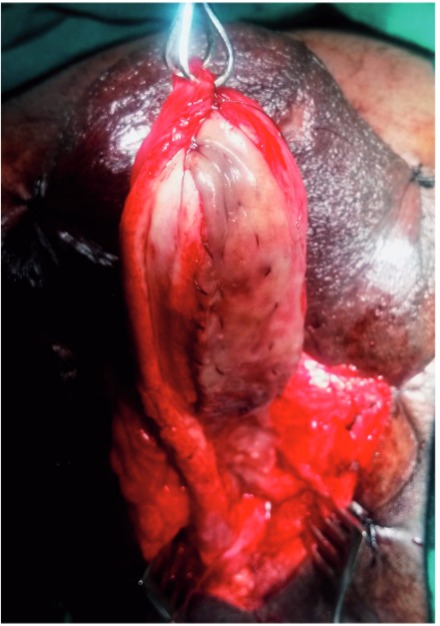
Graft placement covering entire length of stricture and extending into fossa navicularis.

**Figure 5:**
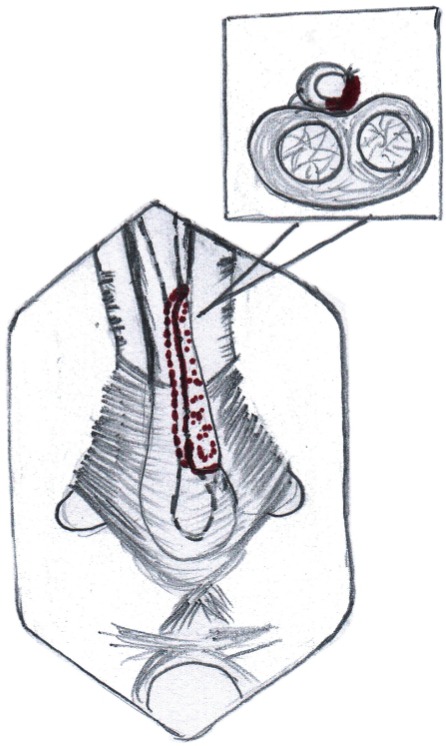
Completed graft anastomosis.

## RESULTS

Twenty patients were included in the study (Table-1). Mean age of patients was 39±7.867 year (range 18 to 56 years). Mean stricture length was 8.5±1.395cm (range 4 to 12cm). Mean operative time was 140±11.337 min (range 120-180 min). The mean postoperative Qmax at the 12-month follow-up was 24±3.162mL/sec (range 18-32mL/sec). None of these patients had any significant post void residual urine. The mean hospital stay was 6.25±1.070 days (range 5-9 days). None of the patients required peri-operative blood transfusions.

**Table 1:** Patient demographics, operative and follow up data.

Total no of patients, *n* =20	Range	Mean ± standard deviation
Age, in years	18-56	39±7.867
Stricture length, in cm	4-12	8.5±1.395
Operative time, in min	120-180	140±11.337
Hospital stay, in days	5-9	6.25±1.070
Post-operative Qmax (12 month), in mL/sec	18-32	24±3.162
Follow-up, in months	12-36	22.7±4.105

Mean follow-up was 22.7±4.105 months (range 12 to 36 months). Treatment was successful in 17 (85%) and failed in 3 (15%). These 3 patients presented with decreased flow rates of<9mL/sec after 1-3 months. VCUG revealed a stricture at the proximal end of the graft in 1 (confirmed by urethroscopy), meatal stenosis in 1, and 1 had recurrent stricture along the whole length of the graft.

Recurrent stricture was treated by DVIU. Meatal stenosis was managed by a meatotomy. The patient who had recurrent stricture of the whole length was planned for revision urethroplasty but he lost follow-up.

Other complications included scrotal oedema in 3 (17.6%), 3 patients (17.6%) had brownish discharge through external meatus and 2 (10%) patients had wound infection (Figure-3). One of these patients of wound infection had an urethrocutaneous fistula, which presented to us 3 weeks after catheter removal. None of the patients in our study had postoperative chordee, diverticulum formation or post void dribble.

## DISCUSSION

BMG augmentation urethroplasty has become the standard of care for long urethral strictures. Whether to place the graft dorsally, ventrally or laterally is controversial. Dorsal placement of graft has advantage of using corporal bodies to provide a secure well vascularized graft bed that helps to prevent protrusion of the graft with resulting pseudo-diverticulum formation. In addition, this spread BMG fixation preserves graft width and hence urethral caliber (10). On the other hand ventral location provides the advantage of ease of exposure and good vascular supply by avoiding circumferential rotation of urethra. Ventral urethrotomy allows the lumen to be clearly delineated, thus enabling the surgeon to identify mucosal edges, measure the size of the plate, carry out water tight anastomosis and if necessary, excise a portion of the stricture and perform dorsal re-anastomosis (3, 11). Barbagli et al., in 2005 published a retrospective study of 50 cases with bulbar urethral stricture where buccal mucosa graft urethroplasty was done. Grafts were placed as ventral, dorsal and lateral onlay in 17, 27 and 6 patients respectively. After a mean follow-up of 42 months, placement of graft into ventral, dorsal or lateral surface of the bulbar urethra showed similar results (12).

Later in 2008, Barbagli et al. showed that the dorsal urethral surface could be easily approached leaving the bulbospongiosum muscle and central tendon of the perineum intact, thus preserving the branches of perineal nerves from surgical injury. The bulbospongiosum muscle is primarily responsible for ejaculation because of its rhythmic contractions with other perineal muscles to expel semen from the urethra. It may also have an important role in expelling urine (8).

Kulkarni et al. published their series of 24 patients in 2009, wherein they described a new technique of one-sided anterior dorsal oral mucosal graft urethroplasty while preserving the lateral vascular supply to the urethra, the central tendon of the perineum, the bulbospongiosum muscle and its perineal innervation and showed a success rate of 92%. They also reported that the factors such as age, cause of stricture, length and prior instrumentation previously said to have influence on any kind of urethroplasty have no effect on the success rate, suggesting that other factors (possibly vascular and neurogenic injury) may play an important role in determining stricture recurrence (9).

In our series of 20 patients overall success was 85%, in a mean follow-up of 22.7 months. We feel that with a single perineal incision and invagination of the penis, adequate exposure of the whole anterior urethra is possible. This approach avoids a separate penile skin incision, making it more cosmetic and also reduces the chances of development of urethrocutaneous fistulas. One-sided dissection of the anterior urethra from the corpora cavernosa allowed us to visualize the urethral lumen with minimal rotation of the urethra. Also, placement of a guide wire/urethral catheter in the urethral lumen acts as a valuable guide while incising the urethra. We were able to avoid creating false passages, especially in very narrow or scarred portions of the stricture by this maneuver. None of the patients on our series had post void dribble following the procedure.

All 3 failures occurred in the early days of the study period. The patient who developed meatal stenosis had a Lichen sclerosus stricture involving the external meatus. The dorsal meatotomy incision that we used in such cases for laying the buccal mucosa on the glans portion of the distal urethra was probably of insufficient depth/width. He was treated by a simple meatotomy, which was sufficient. The cause for stricture at the proximal anastomotic site was similarly due to a failure to achieve mucosa-to-mucosa approximation of the graft and healthy urethra. This was managed by DVIU and the patient remained symptom free till the end of follow-up period. The patient with recurrent pan-urethral stricture was a chronic tobacco chewer and had to quit only 2 months prior to surgery. This could have resulted in a sub-optimal buccal mucosa graft.

Scrotal oedema in 3 (17.6%) was managed conservatively with scrotal support, and oral serratiopeptidase twice a day for 3 days. Three patients (17.6%) had brownish discharge through external meatus that was managed by gently squeezing the shaft from penoscrotal region till the meatus, which subsided in 3 days. This discharge was probably the collected blood that was retained in urethra during dissection. Two patients (10%) had wound infection and they were managed by regular dressings. One of these patients of wound infection had an urethrocutaneous fistula, which presented to us 3 weeks after catheter removal. He underwent reinsertion of suprapubic catheter & regular dressings. A VCUG done 3 weeks later showed resolution of the fistula tract, so the suprapubic catheter was removed. These patients of wound infection had prolonged hospital stay.

Our results are comparable with those published by Kulkarni et al. in 2009, using the same technique (9). Limitations of our study are small number of patients and a short follow-up period of 22.7 months.

## CONCLUSIONS

Dorsolateral placement of buccal mucosa graft for long anterior strictures is minimally invasive, safe and has good outcomes with short to intermediate length of follow-up. Further studies on larger series of patients are necessary to confirm that preservation of the one-sided lateral vascular supply to the urethra and its entire muscular and neurogenic support reduces the incidence of stricture recurrence, post void dribble and ejaculatory dysfunction.
